# Breast cancer patients from the Midwest region of the United States have reduced levels of short-chain fatty acid-producing gut bacteria

**DOI:** 10.1038/s41598-023-27436-3

**Published:** 2023-01-11

**Authors:** Rachel L. Shrode, Jessica E. Knobbe, Nicole Cady, Meeta Yadav, Jemmie Hoang, Catherine Cherwin, Melissa Curry, Rohan Garje, Praveen Vikas, Sonia Sugg, Sneha Phadke, Edward Filardo, Ashutosh K. Mangalam

**Affiliations:** 1 Department of Informatics, University of Iowa, Iowa City, IA 52242 USA; 2 Interdisciplinary Graduate Program in Immunology, University of Iowa, Iowa City, IA 52242 USA; 3 Stead Family Department of Pediatrics, University of Iowa, Iowa City, IA 52242 USA; 4 Medical Scientist Training Program, Carver College of Medicine, University of Iowa, Iowa City, IA 52242 USA; 5 Department of Pathology, Carver College of Medicine, University of Iowa, Iowa City, IA 52242 USA; 6 Department of Microbiology and Immunology, University of Michigan, Ann Arbor, MI 48109 USA; 7 College of Dentistry, University of Iowa, Iowa City, IA 52242 USA; 8 College of Nursing, University of Iowa, Iowa City, IA 52242 USA; 9 Holden Comprehensive Cancer Center, University of Iowa Hospital and Clinics, Iowa City, IA 52242 USA; 10 Department of Internal Medicine, University of Iowa, Iowa City, IA 52242 USA; 11 Department of Surgery, University of Iowa, Iowa City, IA 52242 USA; 12 Division of Hematology, Oncology, and Blood and Marrow Transplantation, Department of Internal Medicine, University of Iowa Carver College of Medicine, Iowa City, IA 52242 USA; 13 Cytonus Therapeutics, Carlsbad, CA USA; 14 University of Iowa, 25 S Grand Ave, 1080-ML, Iowa City, IA 52246 USA

**Keywords:** Cancer, Biomarkers, Diseases, Oncology, Microbiology, Microbial communities, Microbial genetics

## Abstract

As geographical location can impact the gut microbiome, it is important to study region-specific microbiome signatures of various diseases. Therefore, we profiled the gut microbiome of breast cancer (BC) patients of the Midwestern region of the United States. The bacterial component of the gut microbiome was profiled utilizing 16S ribosomal RNA sequencing. Additionally, a gene pathway analysis was performed to assess the functional capabilities of the bacterial microbiome. Alpha diversity was not significantly different between BC and healthy controls (HC), however beta diversity revealed distinct clustering between the two groups at the species and genera level. Wilcoxon Rank Sum test revealed modulation of several gut bacteria in BC specifically reduced abundance of those linked with beneficial effects such as *Faecalibacterium prausnitzii*. Machine learning analysis confirmed the significance of several of the modulated bacteria found by the univariate analysis. The functional analysis showed a decreased abundance of SCFA (propionate) production in BC compared to HC. In conclusion, we observed gut dysbiosis in BC with the depletion of SCFA-producing gut bacteria suggesting their role in the pathobiology of breast cancer. Mechanistic understanding of gut bacterial dysbiosis in breast cancer could lead to refined prevention and treatment.

## Introduction

The global incidence rate of breast cancer has increased substantially since the 1980s, and this heterogenous disease now represents the most diagnosed cancer worldwide^1^. In the United States, breast cancer is responsible for nearly one-third of all cancers diagnosed in women^2^. There are numerous risk factors associated with breast cancer, including both environmental factors (e.g., reproductive history, hormone replacement therapy, obesity, etc.) and familial factors (e.g., family history of genetic mutations in *BRCA1* and *BRCA2*, etc.)^3–5^. However, up to 50% of breast cancer cases cannot be attributed to these known risk factors^6,7^, suggesting that other, unknown factors can also lead to the development of breast cancer. Recently, research has focused on the interactions between the host microbiome and cancer, though the nature of these interactions remains elusive. Although specific bacterial species have been linked to some cancers, such as *Helicobacter pylori* with gastric cancer and *Fusobacterium nucleatum* with colorectal cancers^4^, there is no single bacterium linked with the pathobiology of breast cancer.

The human microbiome consists of many species of bacteria, viruses, fungi, and archaea, and estimates suggest that there are at least as many microbes in and on the human body as human cells^8^. These microbes exist in a complex relationship with the human host and are essential to homeostasis^9^. Bacteria represent the most abundant microorganism that inhabit the human host and interact with the host through manipulation of signaling pathways, hormone release, DNA double-strand breaks, apoptosis and senescence, and inflammation^3,4,10^. Dysbiosis, an atypical microbiome composition, has been correlated with many disease states, including cancer^11^. Evidence suggests that breast cancer patients have bacterial dysbiosis in both the breast microbiome^3^ and the gut microbiome^3,5,10,12–15^.

An association between microbial dysbiosis and breast cancer was reported as early as 1990 in a study that identified significantly different fecal microbial compositions of postmenopausal breast cancer patients (n = 11) compared to healthy controls (n = 7)^12^. More recently, studies have identified significantly different gut microbial compositions in breast cancer patients based on BMI or clinical cancer stage^16–18^. *Goedert *et al*.* demonstrated that postmenopausal breast cancer patients (n = 48) had a significantly lower alpha diversity compared to healthy controls (n = 48)^19^. In contrast, *Zhu *et al*.* observed that postmenopausal breast cancer patients (n = 44) had higher alpha diversity compared to postmenopausal healthy controls (n = 46)^20^. As alpha diversity observes community richness, these studies display contradicting results of the total types of microbes present. These studies show significant variability, but this heterogeneity is not surprising since many factors such as geographical location, weather conditions, population genetics, dietary habits, and green spaces strongly impact gut microbiome composition. Thus, to better understand the role of the microbiome in breast cancer, we need data from multiple geographical regions. Therefore, this study was undertaken to determine whether there is a gut dysbiosis in breast cancer patients from the Midwest region of the United States.

We recruited patients with breast cancer (BC) through the Breast Molecular Epidemiology Resource (BMER) of the Holden Comprehensive Cancer Center (HCCC) and healthy controls (HC) at the University of Iowa. In this pilot study, we report a significant difference in gut microbial composition in BC when compared to race-, body mass index (BMI)-, and sex-matched HC.

## Results

### The composition of the gut microbiome differs between patients with breast cancer and healthy controls

**Figure 1:**
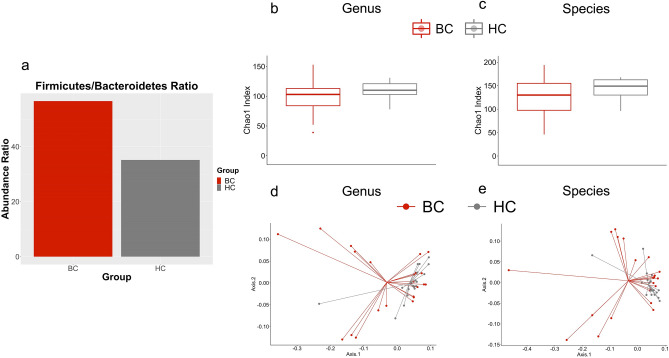
The composition of the gut microbiome differs between patients with breast cancer and healthy controls. (**a**) The Firmicutes/Bacteroidetes Ratio found in patients with BC and HC. The ratios are not significantly different (*p* = 0.06241). (**b**) The Chao1 Index was utilized to measure genera richness. This comparison was not significantly different between BC and HC (*p* = .111). (**c**) This measurement was also utilized to analyze the species level. There was not a significant difference between the two groups at the species level (*p* = .129). (**d**) The Weighted UniFrac distance metric was utilized to analyze beta diversity at the genus level and BC and HC significantly clustered (*p* = 0.011). (**e**) This metric was also utilized to analyze the species level and BC and HC again significantly clustered (*p* = 0.014).

To observe the gut microbiome of the BC (n = 24) and HC (n = 23) cohorts, metagenomic sequencing of the V3-V4 region of 16S rRNA was utilized. After removing subjects with low-quality sequences and a precancerous patient, we had 22 BC patients and 19 HC for further analysis. First, the ratio between Firmicutes/Bacteroidetes was observed as it is considered a marker for dysbiosis^21^. This comparison was performed before filtering or normalization of feature abundances to observe the raw value differences. This ratio was not significantly different between the two cohorts (*p* value: 0.06241) (Fig. 1A). Next the differences between the groups at genera and species levels were analyzed.

In total, 519 species and 340 genera were identified. Of these 519 species, 61 were exclusively found in HC, and 81 were exclusively found in BC patients. Of these 340 genera, 28 were unique to HC, and 49 were unique to BC patients. After filtering, 114 species and 92 genera remained. Alpha diversity of the pre-normalized data was measured with the Chao1 Index; however, it was not significant at the species (*p* = 0.129) or genera (*p* = 0.111) level between BC and HC cohorts (Fig. 1B and C). Shannon diversity was also not significantly different between BC and HC at the species or genera level (*p* = 0.344, *p* = 0.414, respectively). Beta diversity was measured utilizing the Weighted UniFrac distance metric, which compares the microbiomes of each sample by assessing the quantity of the features present while also including the phylogenetic relationships between these features. Beta diversity was statistically significant at the species (*p* = 0.014) and genus (*p* = 0.011) levels, which can also be seen by the distinct clustering of BC and HC into separate groups at both levels (Fig. 1D and E).

**Figure 2:**
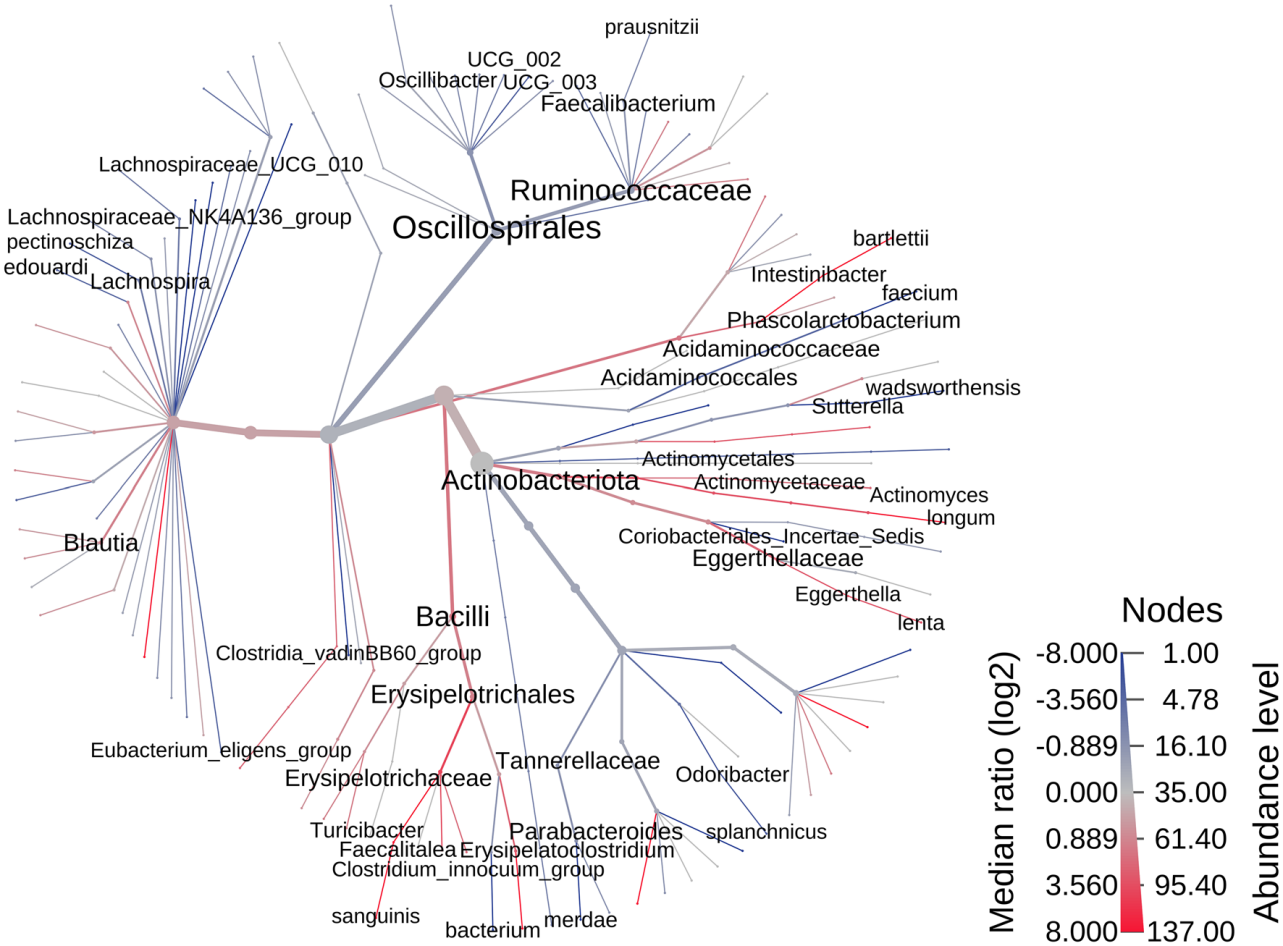
Phylogenetic diversity differences between patients with breast cancer and healthy controls. This heat tree represents the phylogenetic differences between HC and BC. Red indicates a higher abundance in BC compared to HC and blue indicates a lower abundance in BC than HC. This heat tree was created using the MicrobiomeAnalyst web-based interface with updates as of spring 2022^78,79^.

**Figure 3:**
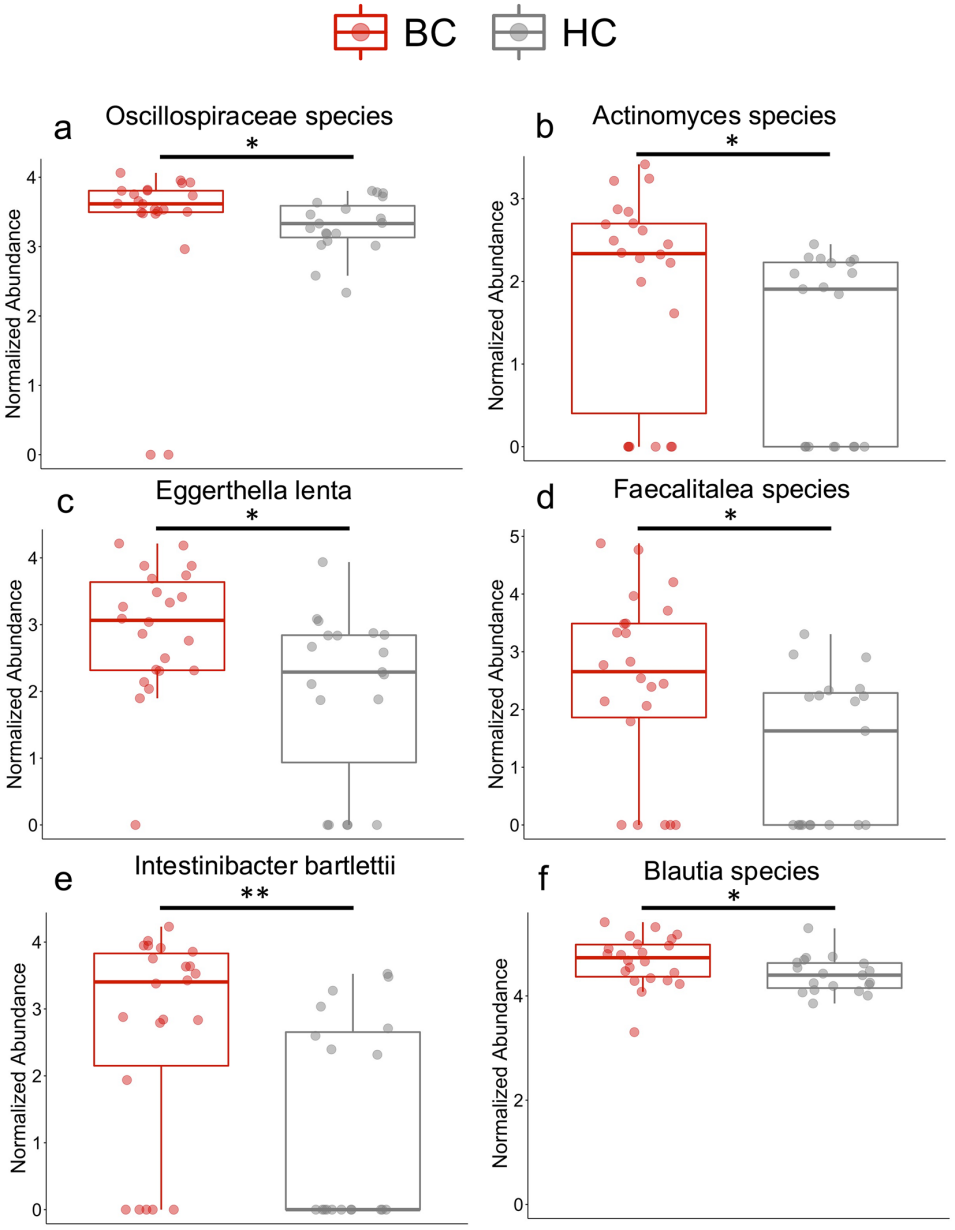
Bacteria significantly increased in patients with breast cancer compared to healthy controls. (**a**–**f**) Based on the Wilcoxon test and the Benjamini–Hochberg procedure, 6 features were significantly higher in abundance in the breast cancer cohort compared to the healthy controls (*p* ≤ 0.05, *q* ≤ .15). Significance: * < 0.05 and ** < 0.01.

**Figure 4:**
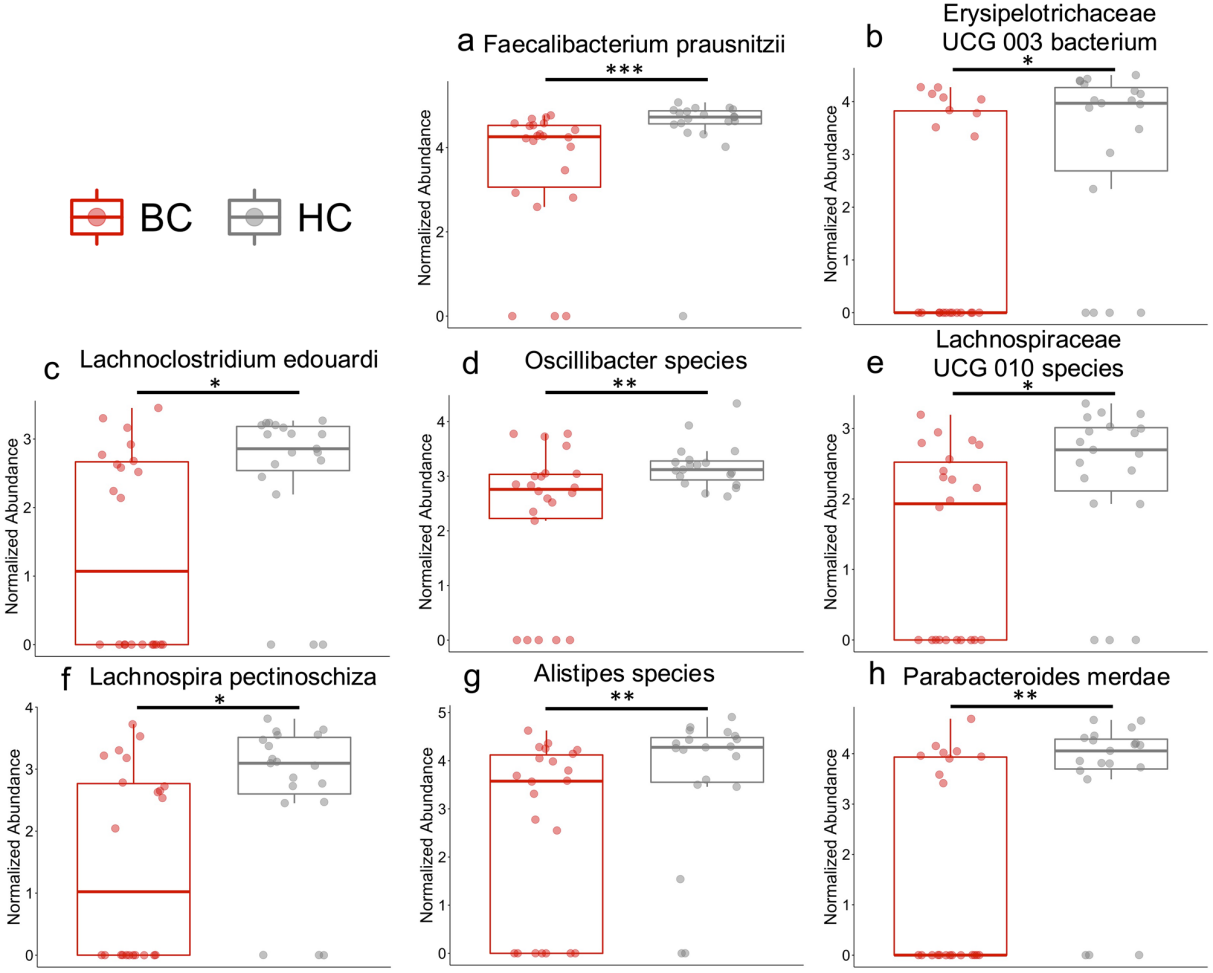
Bacteria significantly decreased in patients with breast cancer compared to healthy controls. (**a**–**h**) Based on the Wilcoxon test and the Benjamini–Hochberg procedure, 8 features weresignificantly lower in abundance in the breast cancer cohort compared to the healthy controls (*p* ≤ 0.05,* q* ≤ 0.15). Significance: * < 0.05, ** < 0.01, and *** < 0.001.

A heat tree was utilized for an overview of the fecal microbiome. This provides a visual representation of the bacterial features enriched or reduced/depleted between groups (Fig. 2). For an overall summary of the most abundant genera, family, and phylum, we included a stacked bar plot at each of these taxa levels in Supplementary Fig. 1. A closer look at these features revealed 16 species that were significantly different between BC and HC based on their normalized log abundance with the Wilcoxon signed rank sum test. Significant features had a *p* value ≤ 0.05 and an adjusted *p* value ≤ 0.15. The notable species that were more abundant in the BC cohort compared to the HC cohort include *Oscillospiraceae species, Actinomyces species, Eggerthella lenta, Faecalitalea species, Intestinibacter bartlettii, and Blautia species* (Fig. 3A–F). The species showing lower abundance in the BC cohort compared to the HC cohort include *Faecalibacterium prausnitzii, Erysipelotrichaceae UCG 003 bacterium, Alistipes species, Oscillibacter species, Lachnospiraceae UCG 010 species, Lachnoclostridium edouardi, Lachnospira pectinoshiza,* and *Parabacteroides merdae* (Fig. 4A–H). A full summary of these results can be found in Supplementary Table 1.

**Figure 5:**
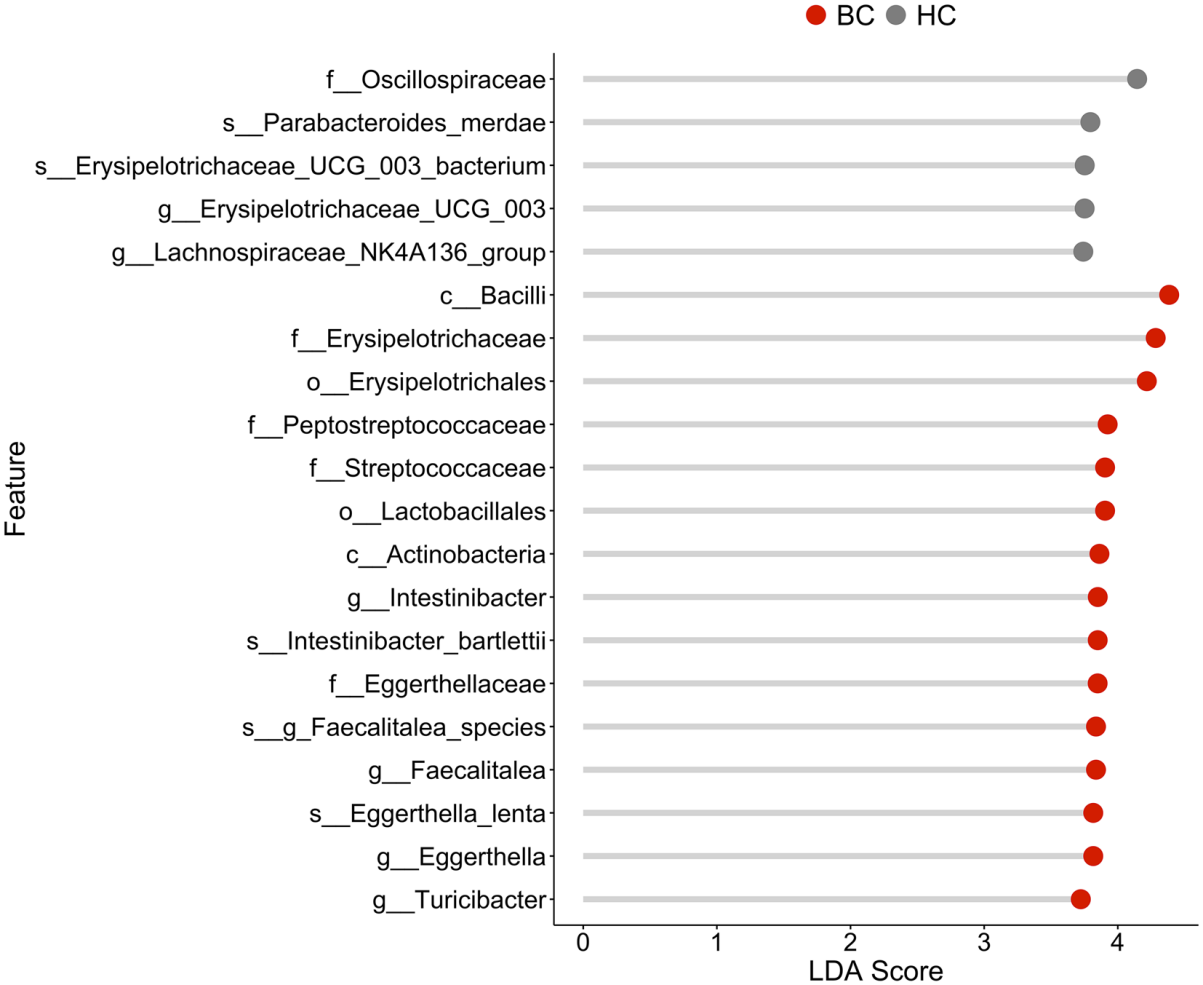
Distinguishing taxa between patients with breast cancer and healthy controls. Top 20 significant features selected by LEfSe analysis. The LDA score indicates the effect size of each feature.

Lastly, we performed Linear discriminant analysis Effect Size (LEfSe) which identifies features distinguishing the two groups by combining statistical test with biological consistency and significance^22^. Using LEfSe, we observed five features with an LDA score of at least three in HC and 15 features with an LDA score of at least three in BC. All the species identified by LEfSe were also identified in the univariate test with the same relationship between HC and BC (Fig. 5).

### Random Forest identifies key species in differentiating between the microbiome of patients with breast cancer and healthy controls

**Figure 6:**
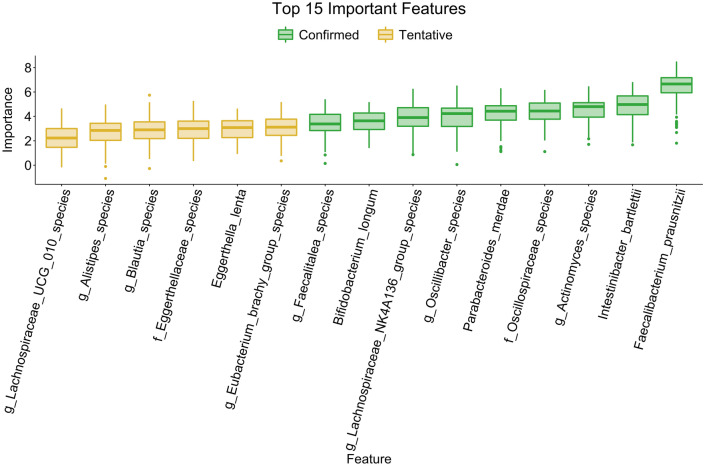
Random Forest identifies key species in differentiating between the microbiome of patients with breast cancer and healthy controls. The random forest machine learning algorithm was utilized to see if the fecal microbiome could differentiate between BC and HC. We utilized a bootstrapped random forest algorithm of 500 trees. Significance was based on the Boruta algorithm with a significance level of 0.01 highlighted in green.

The random forest machine learning algorithm was applied to assess the ability of the fecal microbiome to predict the BC phenotype. Specifically, a bootstrapped random forest of 500 trees was utilized to produce a predictive model based on BC and HC samples. The top 15 features important in identifying which cohort the sample is from are shown in Fig. 6. We then utilized the Boruta^23^ function in R with a significance level of 0.01 to identify the bacterial species important in differentiating between the BC and HC samples. Nine of these 15 features were significant, as seen in green. Seven of these nine significant species were also identified in our univariate analysis as significantly different. The species found to be lower in BC compared to HC in the univariate analysis and were also considered significant in the random forest analysis include *Faecalibacterium prausnitzii, Parabacteroides merdae,* and *Oscillibacter species*. The species that were higher in BC compared to HC in the univariate analysis that were also considered significant in the random forest analysis include *Intestinibacter bartelli, Actinomyces species, Faecalitalea species,* and *Oscillospiraceae species*. Two species were found to be significant based on the random forest analysis but not in the univariate test, *Bifidobacterium longum* and *Lachnospiraceae NK4A136 group species*.

### The functional profile of the gut microbiome differs between patients with breast cancer and healthy controls

**Figure 7:**
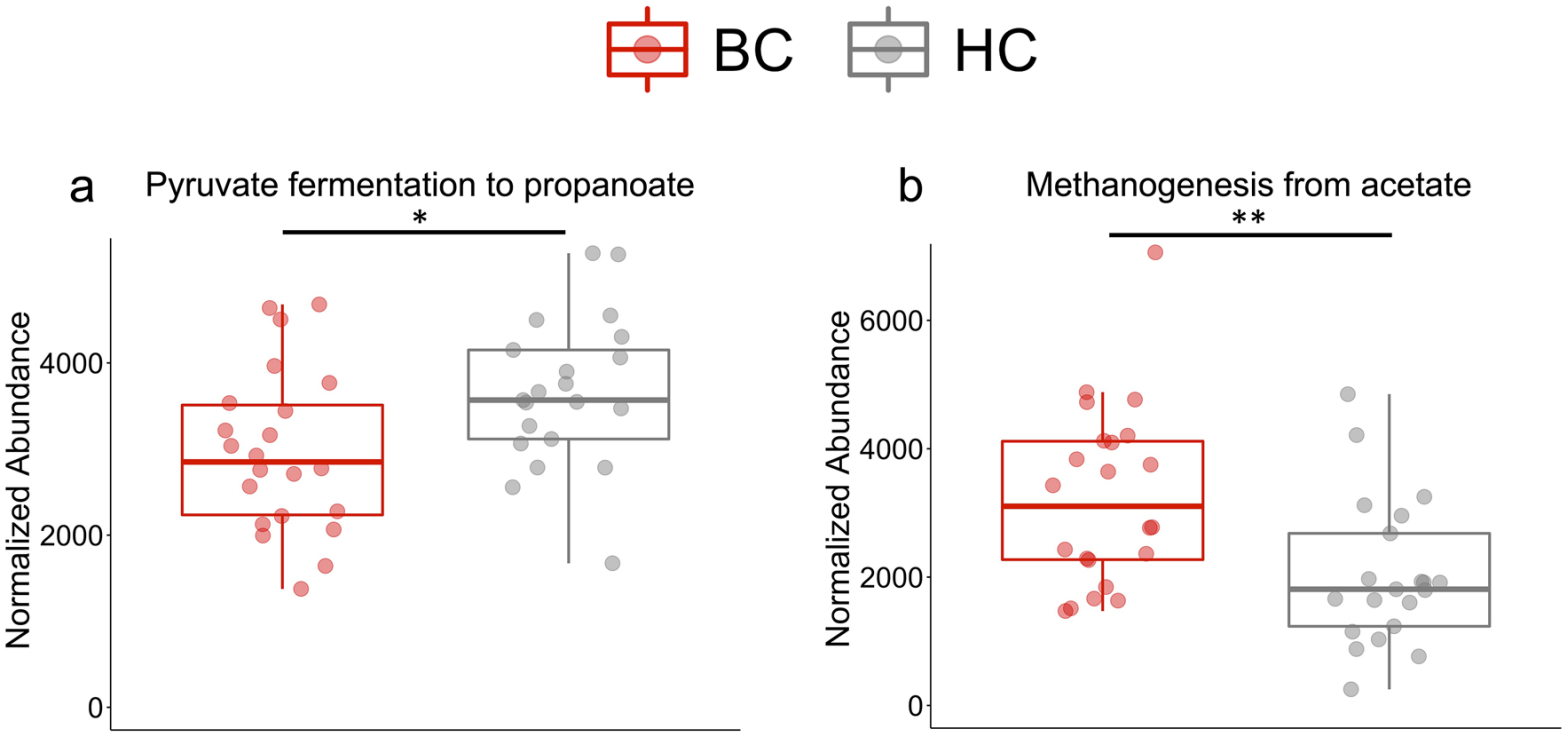
The functional profile of the gut microbiome differs between patients with breast cancer and healthy controls. Based on the Wilcoxon test and the Benjamini–Hochberg procedure, 43 pathways were significantly different between BC and HC. Two pathways were highlighted as they are involved in short chain fatty acid metabolism, (**a**) Pyruvate fermentation to propanoate and (**b**) Methanogenesis from acetate. Significance: * < 0.05 and ** < 0.01.

To estimate the functional profile of the microbiome (metagenome) of our samples, PICRUSt2 (Phylogenetic Investigation of Communities by Reconstruction of Unobserved States) was utilized^24^. This bioinformatics tool analyzed the metagenome of the bacteria in our fecal samples by using the 16S rRNA sequences. In brief, PICRUSt2 estimates the abundance of the gene families in the sample to determine the composition of the metagenome. Through this analysis, 43 statistically significant pathways were identified (Supplementary Table 2). Two of these pathways are involved in short chain fatty acid (SCFA) metabolism: Pyruvate fermentation to propanoate and Methanogenesis from acetate (Fig. 7A and B). Thus, our marker-based functional profiling suggests that BC have distinct functional pathways compared to HC with reduced abundance of pathways involved in the production of SCFA.

## Discussion

The gut microbiome has emerged as a potential factor in the pathobiology of breast cancer. As geographical region and environment play an important role in shaping the individual microbiome, a new region-specific study is required to profile the fecal microbiome in patients with breast cancer. This study is the first to analyze the gut microbiome of breast cancer patients and healthy controls from the Midwest region of the United States for taxonomic composition and predictive functional profiling. Our results demonstrate an altered gut microbiome with reduced SCFA-producing gut bacteria in BC patients.

Our data showing gut dysbiosis in BC is in accordance with prior studies, suggesting a role of the gut microbiome in breast cancer^3,5,10^. Of the eight species showing reduced abundance in our BC cohort, six either produce SCFAs (i.e., *F. prausnitzii, L. pectinoshiza,* and *P. merdae)* or are members of SCFA-producing genera (i.e., *Lachnoclostridium, Alistipes,* and *Oscillibacter*). *F. prausnitzii* embodies 5% of human fecal bacteria^25,26^ and is a major butyrate (C4) producing species in the gut^27–29^. *L. pectinoschiza*, which ferments polygalacturonic acid to formate (C1) and acetate (C2)^30^, also showed reduced abundance in the BC cohort. Finally, *P. merdae*, which produces acetate (C2)^31^, was reduced in the BC cohort.

*Lachnoclostridium, Alistipes,* and *Oscillibacter* are all genera associated with SCFA production. *Lachnoclostridium symbiosum* produces butyrate (C4)^32^, *Alistipes* produces minor amounts of acetate (C2), valerate (C5), propionate (C3), and butyrate (C4)^33^, and *Oscillibacter valericigenes* and *Oscillibacter ruminantium* produce valerate (C5)^34^ and butyrate (C4)^35^, respectively. The species of *Alistipes* and *Oscillibacter* reduced in our BC cohort were unclassified, though they are potential SCFA-producing species due to the properties of phylogenetically similar species. *L. edouardi* is phylogenetically related to *L. symbiosum* (with a 16S rRNA gene sequence identity of 94.26%)^36^, thus suggesting reduction of an additional SCFA producer in our BC cohort.

In the large intestines, SCFAs are the primary bacterial fermentation metabolites of non-digestible carbohydrates. In the human gut microbiome, SCFAs are predominantly acetate (C2), propionate (C3), and butyrate (C4)^37^, but also include formate (C1) and valerate (C5). A change in SCFAs may be associated with various inflammatory conditions (e.g., multiple sclerosis, inflammatory bowel disease, and obesity)^38–40^. In addition, evidence suggests that SCFAs are important for homeostasis through modulation of colonic epithelium integrity, adipocyte lipolysis, and regulation of the immune system^41^. Many of the effects of SCFAs are likely mediated through G-protein coupled receptors GPR43 and GPR41^42^. Specific to breast cancer, SCFAs activate GPR41 and GPR43-mediated signaling pathways in the MCF-7 human breast cancer cell line^43^, and these receptors have demonstrated reduced expression in invasive breast carcinoma and aggressive triple-negative breast tumors when compared to healthy breast tissue^44^.

In contrast, two species associated with SCFA production were significantly enriched in the BC cohort (*Intestinibacter bartletti* and *Faecalitalea species*). *I. bartletti* produces acetate (C2), valerate (C5), and butyrate (C4)^45^, and *Faecalitalea* produces butyrate (C4)^46^. The unclassified species of *Faecalitalea* that was enriched in our BC cohort is a potential SCFA-producing species. It is possible that these SCFA-producing bacteria are dependent on bacteria enriched in the BC cohort and/or SCFA metabolites produced by them feed into inflammatory pathways. Overall, there are more SCFA-producing bacteria significantly depleted in our BC cohort than those enriched. These results suggests that dysbiosis of SCFA-producing bacteria in our BC cohort could be influential to the pathobiology of breast cancer.

We identified 43 significant differentially abundant functional pathways. Interestingly, the pyruvate-fermentation-to-propanoate-I pathway was significantly decreased in the BC cohort, and the methanogenesis-from-acetate pathway was significantly increased in the BC cohort. These changes result in reduction of SCFAs and increased gut methane. Previous studies have associated an increase in gut methane with inflammatory disorders, such as multiple sclerosis and irritable bowel syndrome^47,48^. Microbial SCFAs in the gut increase colonic serotonin production, promoting gastrointestinal motility^49^. A decrease in SCFAs would therefore decrease gut motility, and increased gut methane also slows intestinal transit^50,51^. This decrease in intestinal transit is postulated to increase nutrient absorption, leading to weight gain and obesity^52^. Obesity contributes to systemic inflammation^53^ and increases the risk of developing breast cancer in postmenopausal women^54,55^. Thus, reduced abundance of SCFA-producing bacteria can contribute to breast cancer through induction of inflammation by modulating serotonin.

In addition to SCFA-producing bacteria, *Eggerthella lenta* and a species of the genus *Blautia* were significantly enriched in BC patients compared to HC. Enrichment of *E. lenta* is associated with various inflammatory states, including colitis^56^ and multiple sclerosis^57^. The role of *Blautia* in disease is more controversial, as both depletion and enrichment of this genus has been associated with inflammatory states (e.g.—depletion of *Blautia* in patients with Crohn’s disease^58^, colorectal cancer^59^, and multiple sclerosis^60^; enrichment of *Blautia* in patients with multiple sclerosis^48^ and inflammatory bowel syndrome^61^).

In this study, we aimed to identify the region-specific microbial composition of breast cancer patients in the midwestern United States. It is well established that geography can impact the gut microbiome, as highlighted by a study showing a distinct microbiome composition in individuals from the United States compared to other countries^62^. Relative to our study, *Yatsunenko *et al*.* identified that adults from metropolitan areas of the United States have increased abundance of an unidentified species of *Alistipes* when compared to adults from Malawi and Venezuela^62^. In this study, we identified a decreased abundance of an unidentified species of *Alistipes* in our BC cohort. Due to the increased abundance of an unidentified species of *Alistipes* in healthy adults from the United States, this could represent a United States-specific dysbiosis.

Few studies have investigated the intracontinental region-specific differences of gut microbial compositions at the genus- or species-level. Previously, *Chen *et al*.* analyzed the gut microbial composition of 118 midwestern subjects that varied by sex, race, BMI, age, alcohol use, and tobacco use to establish a midwestern reference population for gut microbiome research^63^. Relevant to our study, they found that the genera *Faecalibacterium, Parabacteroides, Lachnospira,* and *Blautia* represented some of the most prevalent genera in this midwestern healthy population^63^. In our study, we identified that midwestern BC patients had significantly differential abundance of species within these genera. Specifically, our BC cohort displayed decreased abundance of *Faecalibacterium prausnitzii, Parabacteroides merdae*, and *Lachnospira pectinoschiza,* and increased abundance of an unidentified *Blautia* species. Importantly, this region-specific reference population did not show significant difference in alpha- or beta-diversity based on age^63^, suggesting that adult age may play less of a role in microbial diversity in the Midwest region of the United States.

In conclusion, this pilot study displayed dysbiosis in our BC cohort with decreased abundance of SCFA-producing bacteria, decreased production of propionate, and increased conversion of acetate to methane. Our findings support the hypothesis that decreased abundance of SCFA-producing fecal bacteria can contribute to breast cancer pathobiology^64^. We were unable to measure fecal SCFA due to technical difficulties including sample storage. A prior study has shown that SCFAs evaporate from stool at contact with the atmosphere and thus when measuring SCFA levels, samples must be properly stored immediately^65^. Future studies should measure the abundance of SCFAs in stool samples of breast cancer patients to confirm these findings, as previously done in similar studies^15^. A better understanding of the role of gut dysbiosis in breast cancer could lead to refined prevention, treatment, and prognosis.

Limitations to this study include a small sample size, a lack of information on the menopausal status of the healthy controls, a significant difference in cohort ages, and the possible effects of different treatments on the microbiome. Due to the small sample sizes, we were unable to stratify by breast cancer subtypes, but future studies should aim to recruit large enough cohorts to allow for this analysis.

Our BC cohort was significantly older than our HC cohort. Although age could also play a role in the differences in gut microbial composition between our two cohorts, studies are conflicting on the role of age in microbial composition after middle-age. *Ghosh *et al*.* studied a cohort of 2500 individuals and found that elderly individuals had differential abundances of specific taxa in relationship to disease state (i.e., inflammatory bowel disease, colorectal cancer, cirrhosis, type II diabetes, and polyps) when compared to either young adults or middle-aged adults^66^. In contrast, *de la Cuesta-Zuluaga *et al*.* demonstrated that the microbial diversity of healthy women in cohorts from the United States, United Kingdom, and Columbia increases with age and plateaus around 40 years old^67^. In the same study, a cohort from China showed no effect on microbial diversity when stratified by age^67^. These studies suggest that after age 40, age may play a role in the microbiota of some disease states but does not appear to have a significant role in the microbiota of healthy controls. More research in this area is required to better understand the interplay of age, disease, and the microbiome.

We acknowledge chemotherapy and radiation affect the microbiome. In this study, chemotherapy treatment ended at least 145 days prior to sample collection for all BC patients. Currently, it is unknown if chemotherapy has long-lasting effects on the microbiome. Radiation therapy ended at least 31 days prior to sample collection and was targeted to tumors within the breast, which would have spared the gut microbiome. Additionally, we compared the microbiome of BC on chemotherapy (n = 4) vs no chemotherapy (n = 18) and BC on radiation therapy (n = 9) vs no radiation therapy (n = 13) prior to our BC vs HC analysis and found that alpha and beta diversity were not significantly different nor were any species or genera identified as significantly different between these therapies within the BC cohort. Thus, our preliminary investigation suggested that if chemotherapy or radiation had effects on the gut microbiome, those changes had subsided by the time of this analysis and thus BC samples did not need to be further split based on these therapies when compared to HC.

We also acknowledge that hormonal therapy could impact the microbiome. The hormonal therapies that our BC patients were on in this study were selective estrogen receptor modulators (SERMs; i.e. Nolvadex) and aromatase inhibitors (i.e., Arimidex, Aromasin, and Femara). The gut microbiome modulates circulating estrogen levels^68^, and SERMs are toxic to specific gut bacterial species^10^. To our knowledge, the bacteria found to be significant in our study have not been shown to be affected by SERMs. However, the specific effects of aromatase inhibitors on the gut microbiome have not been well established^10^. Future studies should address these shortcomings to strengthen our understanding of the gut microbiome’s relation to breast cancer.

## Methods

### Patient recruitment and demographics

This study was approved by the University of Iowa Institutional Review Board (Iowa City, IA, USA). Patients with BC (n = 24) were recruited from the BMER at the HCCC. Inclusion criteria were a diagnosis of invasive breast cancer of any stage and age 18–90 years old. Exclusion criteria were antibiotic use during sample collection and premalignant or in situ breast disease without concurrent invasive cancer. For BC patients, data was collected on body mass index (BMI), race, age, lymph node status, menopausal status, types and dates of treatments received, and cancer stage. For all BC patients, chemotherapy treatment had ended 145 days or more before sample collection, and the most recent radiation treatment was 31 days or more before sample collection. Of the BC patients, 22 were on hormonal therapies at the time of sample collection.

HC (n = 23) were recruited through the University of Iowa College of Nursing. Inclusion criteria were females ages 18–90. Exclusion criteria were antibiotic or laxative use within four weeks of sample collection and colonoscopy within three months. For healthy controls, data was collected on BMI, race, and age.

One BC patient and four HC were excluded from analysis due to poor sequence quality, and one BC patient was excluded due to a premalignant lesion. This resulted in 22 BC patients and 19 HC. Subject characteristics are described in Table 1.

**Table 1:** Patient and healthy control demographics.

	HC (n = 19)*	BC (n = 22)*		*p* value
Age (mean, SD, in years)	56.10 ± 9.04	67.82 ± 9.56		0.0048
BMI (mean, SD, in kg/m^2^)	25.36 ± 3.92	27.26 ± 4.68		0.1645
Sex	F = 19	F = 22		
	M = 0	M = 0		
Race	White = 19	White = 22		
Menopausal status	Unknown	Post = 20 Pre = 1 Unknown = 1		
Breast Cancer Subtype		HR +	18	
		HER2+	1	
		TNBC	4	
Clinical Stage		0	0	
		1	15	
		2A	6	
		2B	1	
Hormonal Therapy		Nolvadex	8	
		Arimidex	5	
		Aromasin	1	
		Femara	3	

*Sample demographics utilized in analysis. HR = hormone receptor, HER2 = human epidermal growth factor receptor 2, TNBC = triple negative breast cancer.

### Sample collection, DNA extraction and 16S sequencing

Stool samples were collected by patients in Commode Specimen Collection kits (Fisher PA, USA) provided by our laboratory. Stool samples were shipped on ice and received within 24 h of collection. The stool was aliquoted and stored at − 80 °C within 24 h of receipt. For fecal DNA extraction from the samples, we utilized Qiagen DNeasy PowerLyser PowerSoil Kit (Qiagen, Germantown, MD). We followed the manufacturer’s instructions by performing the bead-beating step (PowerLyzer 24 Homogenizer, Omni International, USA). Sequencing of the V3-V4 region of the 16S rRNA was performed as previously described by our laboratory^69^.

### Metagenomic profiling

We processed the raw sequence data of fecal samples utilizing the V3-V4 region of the bacterial 16S rRNA and the DADA2 pipeline^70^. Briefly, we removed the primers, truncated the rest of the sequence based on a Phred quality score of 25, and then denoised the reads. Denoising was used to eliminate inaccurate base calling. Next, paired reads were merged, and chimeras were removed. The remaining sequences produced our amplicon sequence variants (ASVs). To assign taxonomy to these ASVs, the Silva database was utilized (Version 138.1, released March 2021)^71^. After taxonomy assignment, one BC sample and four HC samples were removed due to having a low read depth (i.e., less than 27,000 reads), resulting in 22 BC patients and 19 HC. The remaining samples had 27,000 to 86,874 counts, averaging 64,265 counts per sample.

### Functional profiling

To identify the possible functions of the microbiome, we utilized tools from DADA2 that converted our cleaned sequence data to an ASV table with a corresponding FASTA sequence file. Then, with the use of PICRUSt2^24^, we predicted potential functional pathways.

### Statistical analysis and visualization

For analyses and figure creation, we utilized R (Version 4.0.3)^72^. The alpha diversity, beta diversity, and differential abundance analyses of the present features were performed with in-house scripts that utilized phyloseq^73^, microbiomeMarker^74^, vegan^75^, and ggpubr^76^. Data were normalized by sum scaling to one million reads at the sample level and log (base 10) transforming at the bacteria level. Features with a prevalence of less than 20 and a relative abundance of less than 1e-4 were also filtered out. These cut-offs were chosen to eliminate inaccurate claims of significance due to the absence of the feature in one group and a small presence in the other. In total, 519 species and 340 genera were identified. After filtering, 114 species and 92 genera remained. For our pathway analysis, pathways with a relative abundance threshold less than 0.0001 (percent composition) were filtered out. Alpha diversity was measured utilizing the Chao1 index and Shannon Diversity. For the differential abundance analyses, the Wilcoxon signed-rank test measured significance, and adjusted *p* values were calculated by the Benjamini–Hochberg algorithm. For beta diversity, the Weighted UniFrac distance metric was utilized, and significance of sample clustering was identified by PERMANOVA. LEFSe was performed utilizing the function run_lefse from the microbiomeMarker R package. Random forest was performed with the randomForest^77^ and Boruta^23^ functions in R. More details about the Random Forest analysis can be found in the "Random Forest identifies key species in differentiating between the microbiome of patients with breast cancer and healthy controls" section. LEFSe is a commonly used differential analysis method while Random Forest is a machine learning-based approach. LEFSe identifies the taxa that are significantly increased in abundance in one group compared to the other while also calculating each feature’s effect size. Random Forest, on the other hand, utilizes many decision trees and bagging (majority vote of decision trees) to decide which features help most in differentiating the groups. Thus, applying both very different approaches, and finding the same features in both, allows us to have more confidence in the features identified as significant.

The heat tree was created in MicrobiomeAnalyst^78,79^. The minimum feature count was set to 20, the percent prevalence in each sample was set to 20, and 10% of features were removed based on their inter-quartile range. Total sum scaling was used to normalize the feature data to create the heat tree.

### Ethics approval

This study was performed in accordance with the ethical standards as laid down in the 1964 Declaration of Helsinki and its later amendments or comparable ethical standards. Research specimens and/or clinical data were obtained through the University of Iowa Holden Comprehensive Cancer Center's 'Breast Molecular Epidemiology Resource' (BMER), an Institutional Review Board-approved biospecimen repository and data registry (IRB 201003791).

### Consent to participate

Informed consent was obtained from all individual participants included in the study.

## Supplementary Information

Supplementary Information.

## Data Availability

The 16S microbiome data has been uploaded to the Sequence Read Archive (SRA) under the BioProject ID: PRJNA872152 for free public access. The rest of the data can be made available through contacting the corresponding author.
