# Supplementary material for: Breast cancer patients from the Midwest region of the United States have reduced levels of short-chain fatty acid-producing gut bacteria
Source: Sci Rep. 2023 Jan 11;13:526. doi: 10.1038/s41598-023-27436-3 (PMC9834383; doi:10.1038/s41598-023-27436-3)
Supplement: Supplementary file 1 — Supplementary Information. [file 41598_2023_27436_MOESM1_ESM.pdf]

Supplementary Information

Breast cancer patients from the Midwest region of the United States have reduced levels of short-chain fatty acid-producing gut bacteria

Rachel L. Shrode[1, +], Jessica E. Knobbe[2,3,4, +], Nicole Cady[5,6, +], Meeta Yadav[5,7], Jemmie Hoang[8], Catherine Cherwin[8], Melissa Curry[9], Rohan Garje[10], Praveen Vikas[10], Sonia Sugg[11], Sneha Phadke[12], Edward Filardo[13], and Ashutosh K. Mangalam [1,2,5,7*]

Supplementary Table 1<br>Species	p-value	Adjusted p-value (FDR)	BC vs HC	Identified in random forest
Faecalibacterium prausnitzii	0.000277	0.031578	Decreased	yes
Intestinibacter bartlettii	0.00232	0.13224	Increased	yes
Parabacteroides merdae	0.0036	0.1368	Decreased	yes
Oscillibacter species	0.00791	0.1368	Decreased	yes
Alistipes species	0.00963	0.1368	Decreased	no
Eggerthella lenta	0.0103	0.1368	Increased	no
Lachnoclostridium edouardi	0.0104	0.1368	Decreased	no
Lachnospiraceae UCG 010 species	0.0104	0.1368	Decreased	no
Faecalitalea species	0.0113	0.1368	Increased	yes
Lachnospira pectinoschiza	0.0121	0.1368	Decreased	no
Erysipelotrichaceae UCG 003 bacterium	0.0141	0.1368	Decreased	no
Blautia species	0.0144	0.1368	Increased	no
Oscillospiraceae species	0.0167	0.13842857	Increased	yes
Actinomyces species	0.017	0.13842857	Increased	yes

Supplementary Table 1 The species significantly different between BC and HC.

Supplementary Table 2

**Pathway**	**p-value**	**Adjusted p-value (FDR)**	**BC vs HC**
TCA_cycle_VIII__helicobacter	0.000326	0.108884	increased
dTDP_N_acetylthomosamine_biosynthesis	0.001	0.11133333	increased
beta__1.4_mannan_degradation	0.001	0.11133333	decreased
superpathway_of_2.3_butanediol_biosynthesis	0.00134	0.11189	increased
superpathway_of_L_alanine_biosynthesis	0.00232	0.11743871	increased
superpathway_of__R.R_butanediol_biosynthesis	0.00389	0.11743871	increased
L_arginine_biosynthesis_III__via_N_acetyl_L_citrulline	0.00423	0.11743871	decreased
methanogenesis_from_acetate	0.0046	0.11743871	increased
pyrimidine_deoxyribonucleosides_salvage	0.0046	0.11743871	decreased
superpathway_of_N_acetylglucosamine. _N_acetylmannosamine_and_N_acetylneuraminate_degradation	0.00635	0.11743871	increased
FUCCAT_PWY	0.00688	0.11743871	increased
superpathway_of_demethylmenaquinol_6_biosynthesis_I	0.00688	0.11743871	increased
superpathway_of_demethylmenaquinol_9_biosynthesis	0.00688	0.11743871	increased
X8_amino_7_oxononanoate_biosynthesis_I	0.00688	0.11743871	decreased
tetrapyrrole_biosynthesis_I__from_glutamate	0.00743	0.11743871	increased
queuosine_biosynthesis	0.00743	0.11743871	decreased
biotin_biosynthesis_I	0.00803	0.11743871	decreased
superpathway_of_menaquinol_9_biosynthesis	0.00803	0.11743871	increased
superpathway_of_menaquinol_6_biosynthesis_I	0.00803	0.11743871	increased
superpathway_of_menaquinol_10_biosynthesis	0.00803	0.11743871	increased
pyrimidine_deoxyribonucleotides_de_novo_biosynthesis_IV	0.00803	0.11743871	increased

fatty_acid_elongation____saturated	0.00867	0.11743871	decreased
polyisoprenoid_biosynthesis__E._coli	0.00867	0.11743871	decreased
pyrimidine_deoxyribonucleotides_biosynthesis_from_CTP	0.00867	0.11743871	decreased
glycerol_degradation_to_butanol	0.00936	0.11743871	increased
incomplete_reductive_TCA_cycle	0.0101	0.11743871	decreased
guanosine_nucleotides_degradation_III	0.0101	0.11743871	increased
thiazole_biosynthesis_II__aerobic_bacteria	0.0101	0.11743871	increased
CMP_3_deoxy_D_manno_octulosonate_biosynthesis	0.0109	0.11743871	decreased
gondoate_biosynthesis__anaerobic	0.0109	0.11743871	decreased
mycolate_biosynthesis	0.0109	0.11743871	decreased
oleate_biosynthesis_IV__anaerobic	0.0117	0.1186579	decreased
reductive_TCA_cycle_I	0.0126	0.1186579	increased
peptidoglycan_maturation__meso_diaminopimelate_containing	0.0126	0.1186579	increased
X_5Z_dodecenoate_biosynthesis_I	0.0126	0.1186579	decreased
heme_b_biosynthesis_II__anaerobic	0.0135	0.1186579	increased
stearate_biosynthesis_II__bacteria_and_plants	0.0135	0.1186579	decreased
palmitoleate_biosynthesis_I__from__5Z_dodec_5_enoate	0.0135	0.1186579	decreased
superpathway_of_fatty_acid_biosynthesis_initiation__E._coli	0.0145	0.121075	decreased
pyruvate_fermentation_to_propanoate_I	0.0145	0.121075	decreased
glucose_and_glucose_1_phosphate_degradation	0.0156	0.12708293	increased
purine_nucleobases_degradation_I__anaerobic	0.0179	0.14234762	increased
gluconeogenesis_I	0.0192	0.14913488	decreased

Supplementary Table 2 The functional pathways significantly different between BC and HC.

Supplementary Figure 1

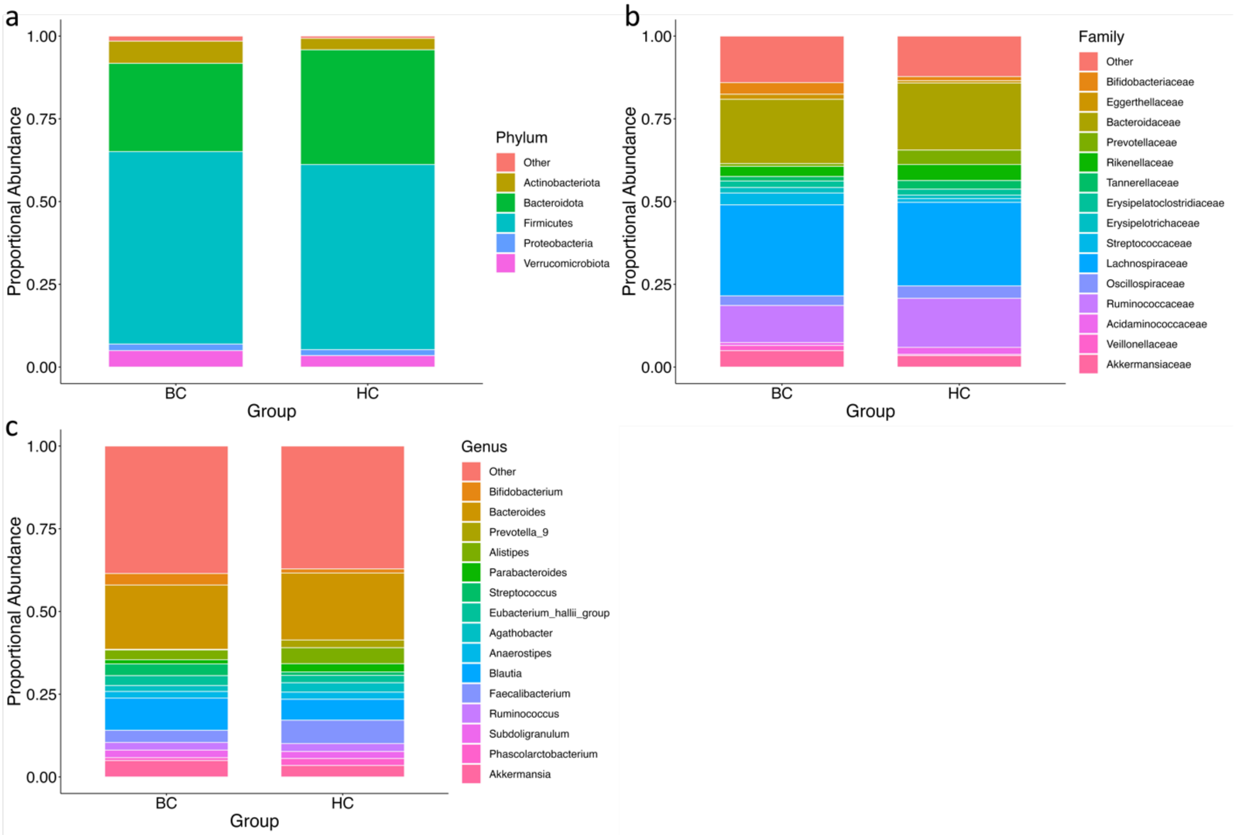

Supplementary Figure 1 The most abundant a) phylum, b) families, and c) genera within the two cohorts.
